# Role of Alphaherpesviruses in Alzheimer's disease pathophysiology

**DOI:** 10.1002/alz70855_103296

**Published:** 2025-12-23

**Authors:** Axel Legrand, Benoit Delatour, Morgane Linard, Catherine Helmer, Oscar Haigh

**Affiliations:** ^1^ Paris Brain Institute (ICM), Inserm, CNRS, Sorbonne University, Paris, Ile de France, France; ^2^ Sorbonne University, Paris, Ile de France, France; ^3^ Sorbonne University, Paris, France; ^4^ Bordeaux Population Health Research Center, Inserm U1219, University of Bordeaux, Bordeaux, France; ^5^ University of Bordeaux, Bordeaux, France; ^6^ University of Bordeaux, INSERM, Bordeaux Population Health Research Center, UMR U1219i, City of Bordeaux, Bordeaux, France; ^7^ CEA, Fontenay‐aux‐Roses, Ile de France, France

## Abstract

**Background:**

Alphaherpesviruses, including Herpes Simplex Virus Type 1 (HSV‐1) and Varicella‐Zoster Virus (VZV), are human neurotropic viruses with a high prevalence worldwide. The relationships between neurotropic Herpesviruses and Alzheimer's disease (AD) have been repeatedly emphasized. Our project is based on the hypothesis that neuro‐invasion of alphaherpesviruses, especially of HSV‐1, could trigger or contribute to the formation of early lesions in AD.

**Method:**

To test our hypothesis, we use a multidisciplinary approach based first on two different animal models for studying the neuropathological consequences of HSV‐1 infection: the cotton‐rat model and the mouse model. Animals were infected in the upper lip and sacrificed at different time points after infection. Histological analyses were conducted to detect viral proteins, microglia, Aß and pTau deposits by immunostaining. Various stains were used to highlight tissue damage. Viral RNAs (lytic or latent phase of HSV‐1) were detected by in situ hybridization (RNAscope). Biochemical assays of Aß/ Tau proteins were performed using the Meso Scale Discovery technology. Another aspect of the project analyses the Shatau cohort, a cohort of human individuals (AD and non demented cases) for which we have serological data for HSV‐1 and VZV, in vivo measurements of Locus Coeruleus integrity, neuroimaging markers of brain accumulation of Aß and pTau proteins, and AD biomarkers in cerebrospinal fluid.

**Result:**

In the different animal models of peripheral HSV‐1 infection we were able to evidence a neuro‐invasion characterized by the presence, in the brainstem, of viral proteins, lytic/latent viral genomes and neuroinflammatory status. In addition, we detected, by immunohistochemistry and biochemical dosages, evidence of abnormal deposition of Aß and hyperphosphorylated tau proteins evocative of AD. Our results in the analysis of the Shatau cohort indicate that, as hypothesized, anti‐viral antibody titrations are increased in AD vs non‐demented control patients. In addition, VZV titers appear surprisingly to be associated with AD biomarkers for the reduction of the Locus Coeruleus integrity and increased in CSF biomarkers, while association with HSV‐1 titers are weaker or absent.

**Conclusion:**

Our results strengthen the hypothesis of a causal connection between infection and neuro‐invasion of alphaherpesviruses and AD neuropathologies.

